# Micro‐CT for the differentiation between live birth and stillbirth: A pilot study

**DOI:** 10.1111/1556-4029.70113

**Published:** 2025-06-26

**Authors:** Giovanni Botta, Tullio Genova, Alessandro Bonsignore, Francesca Buffelli, Federico Davide Mussano, Francesco Lupariello

**Affiliations:** ^1^ A.O.U. Città della Salute e della Scienza—Anatomia Patologica U, Sezione Materno‐Fetale‐Pediatrica Torino Italy; ^2^ Department of Life Sciences and Systems Biology UNITO Turin Italy; ^3^ CIR Dental School, Department of Surgical Sciences UNITO Turin Italy; ^4^ Section of Legal and Forensic Medicine, Department of Health Sciences (DISSAL) University of Genova Genova Italy; ^5^ IRCCS‐Policlinico San Martino Teaching Hospital Genova Italy; ^6^ Fetal‐Perinatal Pathology Unit IRCCS‐Istituto Giannina Gaslini Genova Italy; ^7^ Legal Medine Unit, Department of Public Health and Paediatrics University of Turin Turin Italy

**Keywords:** flotation test, forensic pathology, infanticide, intrauterine fetal demise, live birth, micro‐CT, neonaticide, stillbirth

## Abstract

The distinction between live births and stillbirths is crucial for determining the appropriate legal consequences. Historically, researchers have operated under the principle that an infant's lungs will be filled with air upon death, whereas a fetus's lungs will not. The flotation test for the lungs is the primary method still used in many jurisdictions. However, there are concerns regarding its reliability. For this reason, we conducted a pilot study to evaluate the feasibility of postmortem micro‐computed tomography (micro‐CT) for differentiating between formalin‐fixed aerated and nonaerated lungs. Lung samples from aerated (Group 1) and nonaerated (Group 2) subjects were scanned using micro‐CT. We calculated the gas volume percentage (GV%) in each sample. Group 1 (aerated lungs) showed a mean GV% of 9.52 ± 6.77, while Group 2 (nonaerated lungs) showed a mean GV% of 0.58 ± 0.66. These findings suggest that micro‐CT can qualitatively and quantitatively detect pulmonary aeration and may serve as a valuable tool in forensic investigations involving suspected neonaticide, feticide, and intrauterine fetal demise.

**Highlights**

- Micro‐CT detects and quantifies lung gas, helping distinguish live birth from stillbirth.
- This method could provide a quantitative alternative to the traditional flotation test.
- Aerated lungs: GV% >2.75%; nonaerated: GV% <1.24%.
- Gas volume does not necessarily determine live versus nonlive birth.

## 1 INTRODUCTION

Neonaticide refers to the act of killing an infant within 24 h after birth. It is the most common form of infanticide, with the highest risk occurring on the first day of life [1]. In most jurisdictions worldwide, neonaticide is subject to significantly harsher legal penalties than feticide, making the distinction between the two critically important in medico‐legal investigations [1, 2].

Furthermore, this distinction may carry implications beyond criminal justice; for example, in civil contexts such as inheritance rights or insurance claims, determining whether the infant was born alive may influence legal recognition of personhood [1, 2]. This becomes particularly relevant in cases involving alleged medical malpractice in obstetrics, where establishing whether the infant was born alive or stillborn is crucial to determining the economic compensation owed by insurance providers.

In cases of suspected neonaticide, data obtained from a forensic autopsy can help establish: (1) the gestational age of the infant, to assess potential viability; (2) whether the infant was born alive or stillborn—an important distinction that assists legal professionals in differentiating between neonaticide, feticide, or natural deaths associated with an intrauterine fetal demise (IUFD); and (3) the cause of death [1, 3, 4].

However, establishing whether a neonate was born alive or stillborn remains particularly challenging. This determination is typically based on evidence collected at the scene, witness accounts, and postmortem analysis [3, 4]. Among the methods available, the flotation test for the lungs remains the most commonly used diagnostic tool in many jurisdictions. This test, which dates back to the observation that the lungs of a newborn who had breathed would float in water, is still employed despite its limitations [1, 3, 5]. The test is based on the assumption that aerated lungs float, while nonaerated lungs sink. However, it provides only indirect evidence of respiration.

Importantly, the flotation test does not directly demonstrate the presence of air in the lungs; rather, it indicates the presence of material with a density lower than water. As such, the test is susceptible to false positives due to the presence of other low‐density substances such as decomposition gases or air introduced during resuscitative efforts. This aspect must always be considered when interpreting a positive result—especially in legal proceedings where a diagnosis of live birth may carry severe consequences for the accused [1, 5, 6, 7].

In this context, alternative radiological methods may offer further objective criteria for lung aeration. Computed tomography (CT), widely used in clinical diagnostics since the 1970s, enables the differentiation of tissues based on radiodensity, with water set at 0 and air at −1000 Hounsfield units [1, 3, 5, 8]. This principle allows the identification of aerated versus nonaerated tissues, a concept that has been extended to postmortem investigations. Although conventional postmortem CT has demonstrated effectiveness in identifying lung gas patterns [1, 3], its resolution is limited to voxel sizes of about 1 mm^3^. In contrast, micro‐CT offers submicron resolution and has recently gained attention in various biomedical applications [9, 10]. Despite its potential, micro‐CT remains nonstandard in clinical and forensic settings due to long acquisition times, the need for mechanical stabilization, and the limitation to small sample sizes [9, 10].

Considering the above, we propose a pilot study to explore a new method for qualitatively and quantitatively distinguishing between aerated and nonaerated lungs using micro‐CT. In this manuscript, we highlight the technical aspects related to the use of micro‐CT in this field, as this application has not yet been addressed in the existing literature [7].

## 2 MATERIALS AND METHODS

**FIGURE 1:**
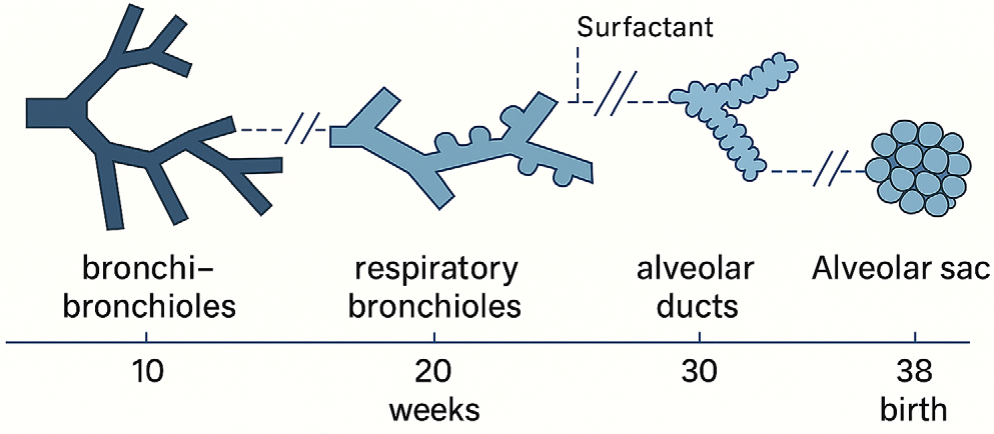
Graphical representation of the development of alveolar spaces as gestational weeks progress.

We analyzed two groups of lung samples. Group 1 consisted of the lungs from five neonates and five adults, which were aerated (refer to Table 1). Group 2 included ten fetuses (Table 2), free of malformations at the level of the lung structure, with a gestational age between 24 and 41 weeks; therefore, characterized by both the saccular phase and the alveolar phase as shown in the following figure (see Figure 1).

**TABLE 1:** Summary of the main characteristics of Group 1.

Sample	Age	Cause of death	Gestational age at birth	Execution of artificial ventilation	Presence of malformations/abnormalities
A1	76 years	Pulmonary thrombosis	Not applicable	Not applicable	Not applicable
A2	50 years	Traffic accident	Not applicable	Not applicable	Not applicable
A3	54 years	Traffic accident	Not applicable	Not applicable	Not applicable
A4	63 years	Acute myocardial infarction	Not applicable	Not applicable	Not applicable
A5	62 years	Ventricular arrhythmia	Not applicable	Not applicable	Not applicable
A6	1 day	Acute respiratory insufficiency due to pulmonary immaturity	26	Yes	No
A7	1 day	Sepsis	38	Yes	No
A8	A few hours	Fetomaternal transfusion	40	No	No
A9	1 day	Nontraumatic cerebral hemorrhage	28	Yes	No
A10	A few hours	Nontraumatic cerebral hemorrhage	27	Yes	No

**TABLE 2:** Summary of the main characteristics of Group 2.

Sample	Gestational age	Cause of stillbirth	Time that lasted between the demise and the autopsy	Execution of artificial ventilation	Presence of malformations/abnormalities
NA 1	38 + 6	Placental abruption	24 h	No	No
NA 2	33	Fetomaternal transfusion	24 h	No	No
NA 3	33	Fetomaternal transfusion	5 days	No	No
NA 4	39	Umbilical knots	5 days	No	No
NA 5	32	Intrauterine growth restriction due to placental causes	24 h	Yes	No
NA 6	36	Placental abruption	24 h	No	No
NA 7	35 + 2	Umbilical knots	5 days	No	No
NA 8	24	Sepsis due to intrauterine bacterial infection	24 h	No	No
NA 9	41 + 1	Umbilical knots	24 h	No	No
NA 10	38	Fetomaternal transfusion	24 h	No	No

For each case included in the study (see Tables 1 and 2), we collected a set of clinical and pathological data and recorded them in a dedicated Excel spreadsheet. The data collection process differed slightly between the two study groups, based on the characteristics of the subjects.

- Group 1 (aerated lungs, five adult individuals and five neonates):

- For the adult cases, we recorded the age at death and the documented cause of death.
- For the neonatal cases, we recorded the following variables: age at death, cause of death, gestational age at birth, whether artificial ventilation had been performed (yes/no), and the presence or absence of malformations or structural abnormalities of the lungs (yes/no).

b Group 2 (nonaerated lungs, fetal cases):

- For each fetus, we recorded the gestational age at birth, the cause of stillbirth, the time interval between the intrauterine demise and the autopsy, whether artificial ventilation had been attempted after delivery (yes/no), and the presence or absence of pulmonary malformations or abnormalities (yes/no).

In all cases, a lung tissue sample was obtained from the apex of the right lung. Each sample measured approximately 2 cubic centimeters. All lungs had been entirely fixed in formalin prior to the sampling procedure. The apical region was chosen because it is typically the most aerated part of the lung, thus maximizing the potential to detect differences in gas distribution between the two groups.

Each sample was scanned using micro‐CT (X‐ray microtomography and SkyScan1172 Bruker) without the application of any staining compounds. The acquisition time was set to 2 h. Subsequently, we used specialized software (CTVox) for the analysis.

In the first step of our analysis, we examined the scans of the samples to determine whether the micro‐CT could identify gas within the formalin‐fixed lung samples. We calculated the radiodensity, expressed on the Hounsfield scale, using CTVox.

The second step involved identifying a quantitative measure that could serve as an indicator of the gas present in each sample, helping us distinguish between aerated and nonaerated lungs. To quantify the gas volume present in each sample, we used the software CTVox to calculate a value representing the total gas volume about the entire volume of the sample. We determined the percentage of gas volume (GV%) using the following formula: GV% = gas volume (GV) divided by the entire volume (EV) of the sample multiplied by 100; GV% = (GV/EV) × 100. We calculated the mean and standard deviation of GV% values for each group using Excel statistical formulas.

In the third step, we conducted a preliminary comparison between Group 1 and Group 2 using the previously mentioned quantitative data.

## 3 RESULTS

Table 1 details the individual characteristics of the cases included in Group 1 (aerated lungs), comprising five adults and five neonates. For adult cases, data reported include age at death and certified cause of death. For neonatal cases, the dataset includes age at death, cause of death, gestational age at birth, artificial ventilation status (performed: yes/no), and the presence or absence of pulmonary malformations or structural abnormalities.

Table 2 provides the corresponding data for Group 2 (nonaerated lungs), consisting of ten fetal cases. For each fetus, we documented gestational age, cause of intrauterine death, time interval between fetal demise and autopsy, artificial ventilation status postdelivery (yes/no), and the presence or absence of morphological abnormalities affecting the lungs.

**FIGURE 2:**
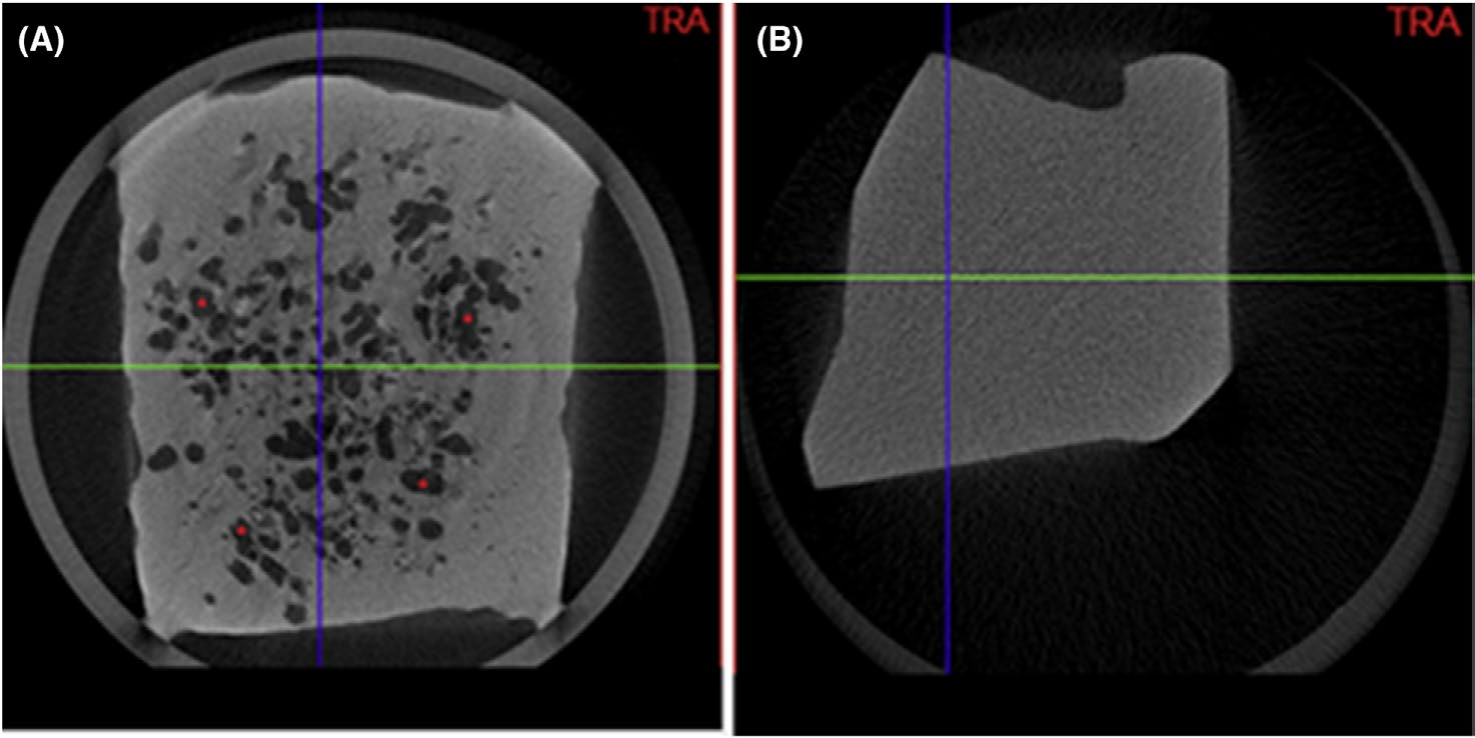
Micro‐CT scans of a Group 1 (A) sample and Group 2 sample (B); in A, aerated areas are visible (*).

Figure 2 shows that the scans could qualitatively identify gas in formalin‐fixed lung samples. This was also confirmed by calculating the radiodensity of each sample using the CTVox software and the Hounsfield scale. Nonaerated samples showed little or no gas, while aerated samples contained gas (Figure 2).

**FIGURE 3:**
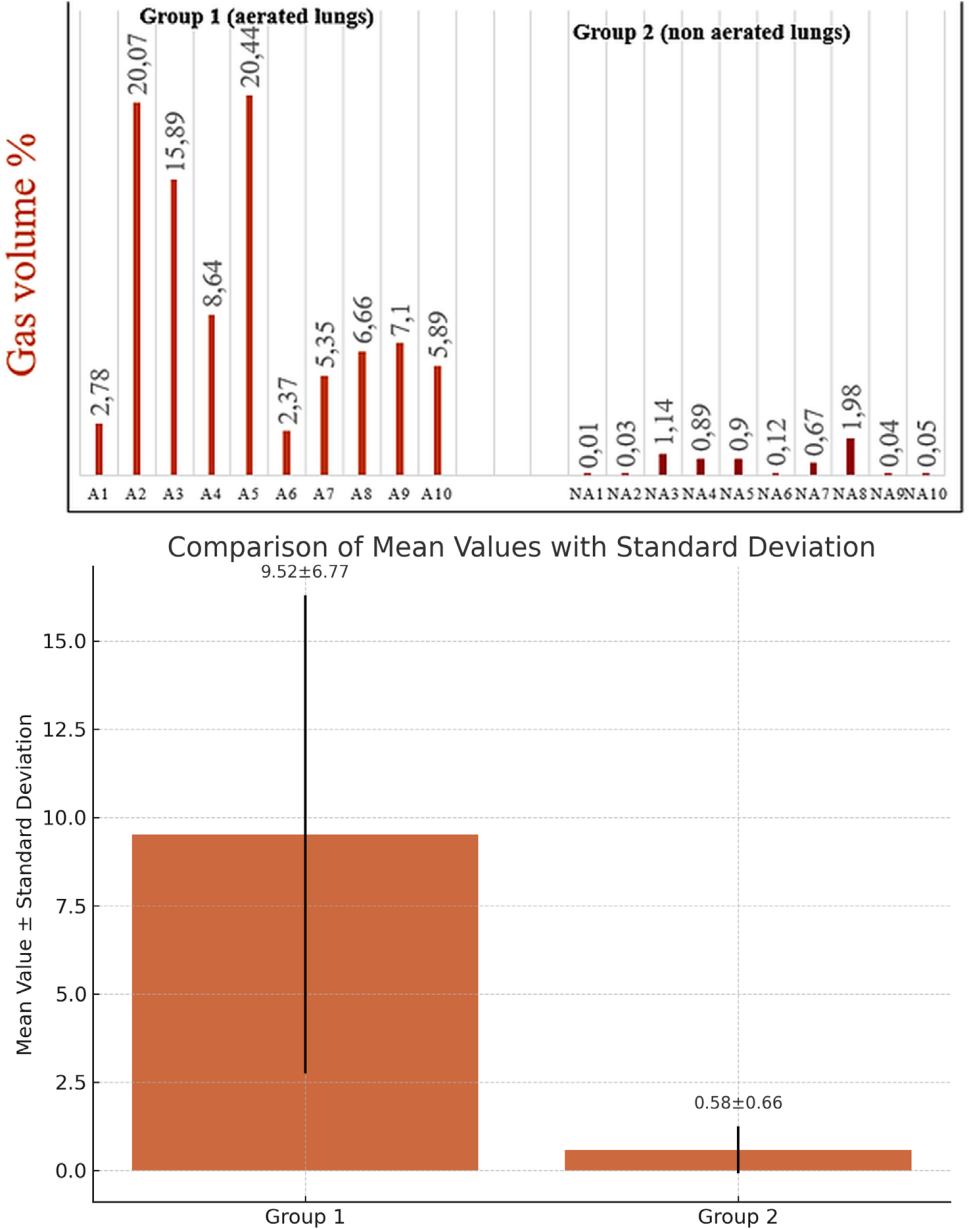
Graphic representation of the GV% of each sample of both groups, means, and standard deviations. A, aerated; NA, nonaerated.

For each sample, we calculated GV%. This value directly represents the gas volume relative to the entire volume of each sample. Thus, the result from the GV% formula is independent of the overall sample volume, which is essential for enabling direct comparisons between samples of different sizes. The results of the GV% formula are graphically represented in Figure 3. The means and standard deviations of GV% values were respectively: 9.52 ± 6.77 for Group 1 and 0.58 ± 0.66 for Group 2 (see Figure 3).

Based on the mean and standard deviation values for Group 1 (mean = 9.52; SD = 6.77), none of the individual samples fell below the lower bound of the mean minus one standard deviation (i.e., 2.75). This suggests that the distribution, although dispersed, remains entirely above this threshold, indicating an overall shift of the dataset toward higher values.

According to the mean and standard deviation values for Group 2 (mean = 0.58; SD = 0.66), none of the individual samples exceeded the upper bound defined by the mean plus one standard deviation (i.e., 1.24). This indicated that the dataset was not only characterized by low central values but also tightly clustered within a narrow range, with no extreme high values observed. However, the standard deviation was slightly influenced by five out of ten cases showing lung gas volumes above the group mean (i.e., >0.58%) but still within the upper bound of 1.24%. The remaining five cases had gas volume percentages ranging from 0.01% to 0.12%, markedly lower than the group mean. A detailed review of the five higher cases suggested potential postmortem factors contributing to gas presence (see Table 2). In one case, the fetus underwent artificial ventilation after expulsion from the uterus. In three cases, autopsies were performed 5 days after fetal demise, allowing time for bacterial proliferation and gas formation. In the final case, fetal death was attributed to an infectious process, which may also have led to intrapulmonary gas accumulation through bacterial activity (Table 2).

## 4 DISCUSSION

In our study, micro‐CT scans successfully distinguished aerated lungs from nonaerated fetal lungs, as supported by radiodensity values (Figure 2). GV% allowed standardized comparisons across samples, with Group 1 showing higher and more dispersed values (9.52 ± 6.77), and Group 2 showing lower and tightly clustered values (0.58 ± 0.66) (Figure 3). All Group 1 values were above 2.75%, confirming a shift toward higher gas content. Group 2 values remained below 1.24%, though five cases slightly above the mean were linked to postmortem factors such as artificial ventilation, delayed autopsy, or infection (Table 2). These findings support the potential of micro‐CT as a reliable tool for assessing pulmonary aeration in forensic contexts.

Building on these results, this pilot study further highlights that micro‐CT is a promising method for differentiating between aerated and nonaerated lungs. Notably, the lengthy scanning process, the need for mechanical stabilization, and the capacity to analyze small samples do not represent limiting factors for this type of analysis. Additionally, micro‐CT can examine samples without requiring staining compounds, which eliminates the need for complex processing or significant modifications to the samples. Furthermore, this technique can study formalin‐fixed samples, allowing for later revisions. The ability to analyze lungs from already formalin‐fixed and stored organs is a significant advantage compared to conventional CT, as it enables case revision after autopsies have been completed. The preservation of air spaces occurs because formalin, being an aqueous solution, does not dissolve or compress existing gas bubbles within tissues. Instead, it fixes the surrounding tissue structure, effectively “locking in” any air that was present at the time of fixation. Moreover, formalin solution inhibits the proliferation of most microorganisms by alkylating proteins and bonding with the nitrogen atoms in purine bases. This action reduces the postmortem production of gas [11].

## 5 CONCLUSIONS

In conclusion, this analysis indicates that micro‐CT imaging can detect the presence of gas in the lungs and calculate gas volume percentages, which can be useful for comparison. Our sample size is still too small to establish a definitive cut‐off value for scientific and forensic applications. Similar considerations also apply to understanding the potential effects of death causes and postmortem changes on lung gas content. These preliminary findings provide a basis for future research to determine how micro‐CT might differentiate between live births and stillbirths in forensic practice.

## CONFLICT OF INTEREST STATEMENT

The authors have no conflict of interest to state.
